# Nomograms and an inflammation model predict immunotherapy prognosis in unresectable hepatocellular carcinoma: from survival and response

**DOI:** 10.3389/fonc.2026.1782572

**Published:** 2026-09-10

**Authors:** Er-lei Zhang, Peng Ma, Wen-jun Zhao, Jia-jia Du

**Affiliations:** 1 Hepatic Surgery Center, Tongji Hospital, Tongji Medical College, Huazhong University of Science and Technology, Wuhan, China; 2 Shanxi Bethune Hospital, Shanxi Academy of Medical Sciences, Third Hospital of Shanxi Medical University, Tongji Shanxi Hospital, Taiyuan, China

**Keywords:** CAR, hepatocellular carcinoma, immunotherapy, NLR, objective response rate

## Abstract

**Background:**

Screening out individuals who could benefit from immunotherapy is important in clinical practice.

**Methods:**

Clinical records of 199 individuals with unresectable HCC who underwent immunotherapy were retrospectively analyzed. Based on the effect of treatment, individuals were classified into two groups: the response group and the non-response group. NLR, LMR, and CAR were obtained before treatment. Kaplan–Meier method and Cox proportional risk regression model were used for survival analysis, and nomograms were established. A clinical prediction model was developed using logistic regression.

**Results:**

The cutoff values of NLR, LMR, and CAR were 5, 1.8, and 0.105, respectively. Individuals with NLR <5, LMR ≥1.8, and CAR <0.105 had higher ORR (43.09% vs. 13.16%, p < 0.001; 57.89% vs. 25.47%, p < 0.001; 41.49% vs. 22.86%, p < 0.01). In the multivariate analysis, it was determined that NLR and CAR independently served as prognostic factors for both PFS and OS. A combined parameter derived from these independent prognostic factors for ORR yielded an area under the curve (AUC) value of prediction probability of 0.825. Notably, the predictive performance of the model surpassed that of NLR (AUC = 0.794), LMR (AUC = 0.732), and CAR (AUC = 0.774).

**Conclusion:**

NLR, LMR, and CAR are potential predictive biomarkers of immunotherapy efficacy in individuals with advanced HCC.

## 1 Introduction

Hepatocellular carcinoma (HCC) ranked as the sixth most prevalent cancer globally, accounts for 75%–85% of all primary liver cancers (1). China accounts for over 50% of new cases reported worldwide, primarily attributed to the hepatitis B virus (HBV) (2). The majority of individuals with HCC receive a diagnosis when the disease has already progressed to an advanced stage, accompanied by chronic liver disease, making surgical treatment unfeasible. Even when detected in its early stages, there is still a recurrence rate of around 70% within five years following radical resection, with a reported 5-year progression-free survival (PFS) rate of only 24% (3). Tyrosine kinase inhibitors, such as sorafenib, have improved the survival of HCC individuals to a certain extent; however, the overall survival (OS) was much lower than clinical expectations. The Chinese government has implemented various strategies to manage cancer and mitigate associated risk factors, resulting in remarkable accomplishments. However, there are still significant hurdles that need to be addressed. Healthy China 2030 aims to increase the 5-year OS rate of liver cancer by 15%.

In recent years, the advent of immune checkpoint inhibitors (ICIs) has brought about a transformative shift in the treatment landscape for unresectable HCCs. ICIs not only improve the response effect and prolong survival but also exhibit a superior safety profile in comparison to targeted drugs (4). Compared with sorafenib, the IMbrave150 trial showed that the combination of atezolizumab and bevacizumab prolonged OS (19.2 vs. 13.4 mo, hazard ratio [HR] 0.66, 95% confidence interval [CI] 0.52–0.85, p < 0.05; p = 0.0009) and PFS (6.9 vs. 4.3 mo, HR 0.65, 95% CI 0.53–0.81, p = 0.0001) (5). However, due to complex liver immune function and individual differences in the maintenance of immune tolerance and immune activation balance, not all individuals with unresectable HCC can benefit from targeted therapy and immunotherapy (6). Given the high-cost burden linked to ICI therapy and the life-threatening immune-mediated toxicity observed in approximately 2%–4% of individuals, there is an urgent need to determine predictive biomarkers to help identify individuals most likely to benefit from targeted therapy and ICI therapy (7). However, unlike other malignancies, ICI combination therapy of HCC is mainly empirical, and there are no biomarkers that can effectively predict drug resistance or efficacy (8). Furthermore, biomarkers commonly used to predict immunotherapy response in various other types of tumors, such as PD-L1 expression (9) and tumor mutation burden (TMB) (10), continue to be a subject of debate when applied to HCC. In recent times, next-generation sequencing (NGS) has identified WNT/β-catenin mutations as potential markers for predicting resistance to ICIs in individuals with unresectable HCC. Nevertheless, the widespread clinical application of NGS is hampered by its substantial cost and intricacy (11).

As an important hub connecting intestinal contents and systemic circulation, the liver is rich in various antigens related to diet and environment, as well as microorganisms derived from the gut. Therefore, the liver has a strong immune tolerance. Meanwhile, the liver also retains immune surveillance capabilities against pathogenic infections and malignant cells. Hence, it must maintain a delicate balance between immune tolerance and immune activation (12). Tumor-infiltrating lymphocytes are part of the anti-tumor inflammatory response, while neutrophils and monocytes inhibit anti-tumor immune cell function and promote neovascularization by secreting vascular endothelial growth factor (VEGF), which is linked to tumor angiogenesis, immune escape, and metastatic disease (13). C-reactive protein (CRP) may inhibit the proliferation and effector function of activated CD4+ and CD8+ T cells. Inflammation is critically involved in the pathogenesis of HCC (14) and participates in the mechanism of ICIs. Systemic inflammatory indicators such as neutrophil-lymphocyte ratio (NLR) (15), lymphocyte-monocyte ratio (LMR) (16), and CRP-albumin ratio (CAR) (17) have been confirmed as biomarkers for HCC prognosis (18). These inflammatory indicators have the advantages of being noninvasive, inexpensive, and readily available in routine practice (19). However, their value in predicting the efficacy of targeted therapy and ICIs in HCC remains unelucidated.

Therefore, in the current research, the predictive value of NLR, LMR, and CAR was assessed as biomarkers of immunotherapy efficacy in individuals with unresectable HCC.

## 2 Material and methods

### 2.1 Enrollment of patients

The clinical information of 243 individuals was retrospectively collected. These individuals encompassed those with unresectable HCC confirmed by pathology or imaging who received targeted therapy and immunotherapy at Tongji Hospital, Huazhong University of Science and Technology from 2019 to 2022. This study adhered to the principles outlined in the Helsinki Declaration and received approval from the Ethics Committee of the Tongji hospital (TJ-IRB20230866). Considering the retrospective nature of the study, the committee waived the necessity for informed consent from all individuals.

**Figure 1:**
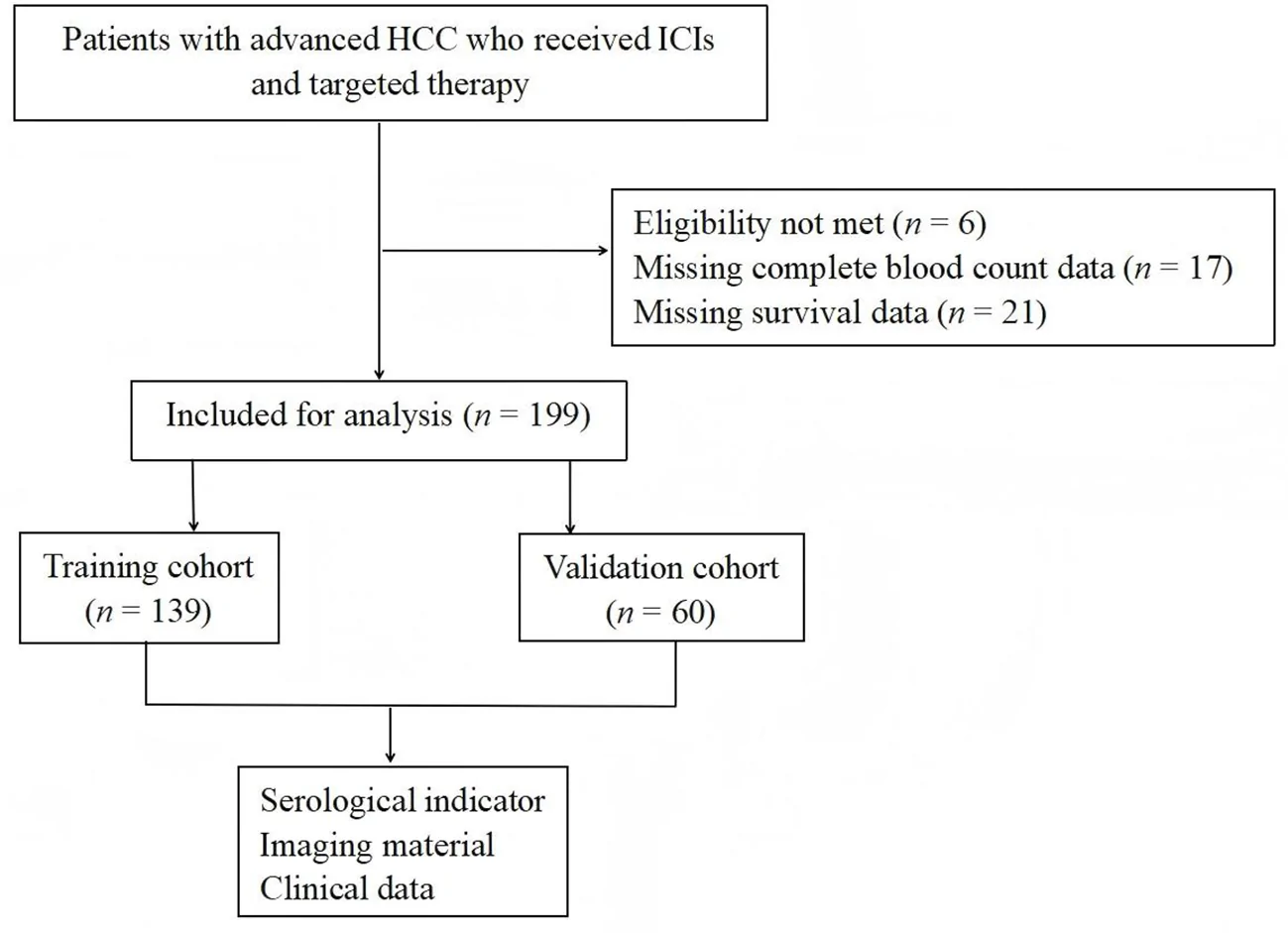
Study population after excluding patients not eligible for the final analysis. HCC, hepatocellular carcinoma; ICI, immune checkpoint inhibitors.

Out of these, 44 individuals were excluded due to incomplete data, leaving a total of 199 individuals in the final analysis (**Figure 1**). The enrolled patients were randomly divided into the training cohort and the validation cohort at a ratio of 7:3. The medical record features collected included patient demographics, HCC etiology, Eastern Cooperative Oncology Group (ECOG) performance score, blood indicators (routine blood, liver function, and alpha-fetoprotein [AFP]), imaging data (maximum tumor diameter, number of metastases, vascular lymph node metastases, cirrhosis, and fatty liver), Child–Pugh class, albumin-bilirubin (ALBI) grade, Barcelona Clinic Liver Cancer (BCLC) stage, and type of targeted therapy and immunotherapy, and whether to receive local therapy.

The inclusion criteria were described below: 1) age 18–75 years; 2) expected survival >6 months, and ECOG score 0–1; 3) Child–Pugh 5–7 points; 4) BCLC stage: B and C; 5) neutrophil ≥1.5 × 10^9^/L, lymphocyte ≥0.5 × 10^9^/L, hemoglobin ≥90 g/L, and platelet ≥75 × 10^9^/L; 6) aspartate aminotransferase and alanine aminotransferase ≤3× the upper limit of normal value (ULN) and total bilirubin ≤3× the ULN; 7) international normalized ratio ≤1.5× the ULN; 8) creatinine ≤1.5* the ULN; 9) prior radiation therapy (RT), transcatheter arterial chemoembolization (TACE), hepatic arterial infusion chemotherapy (HAIC), and radiofrequency ablation (RFA) are permissible; and 10) follow-up targeted, surgical, RT, TACE, HAIC, and RFA are permissible.

The exclusion criteria were as follows: 1) previous chemotherapy, targeted therapy, immunization, surgery, and other systematic treatments; 2) individuals received antibiotics, hormones, immunosuppressants, aspirin, and atorvastatin 1 month before or during targeted therapy and immunotherapy; 3) acute inflammation, diabetes, hyperlipidemia, kidney disease, rheumatic disease, thyroid disease, autoimmune disease, or immune deficiency; 4) distant metastasis (except lymph node metastasis); and 5) malignancy with other sources.

### 2.2 Treatment procedure

ICIs were administered intravenously every 3 weeks for 1 year or until disease progression and intolerable adverse reactions occurred. Blood samples were collected before each cycle of ICI injection. Tumor size alterations were evaluated using enhanced computed tomography or magnetic resonance imaging every six weeks, employing both mRECIST and RECIST 1.1 criteria. Based on the treatment effect, the individuals were classified into two groups: the response group (R), which consisted of complete response (CR), partial response (PR), and stable disease (SD) individuals with PFS >6 months, and the non-response group (NR), which consisted of progressive disease (PD) and SD individuals with PFS ≤6 months. Adverse reactions were evaluated using the Common Terminology Criteria for Adverse Events, version 5.0. If a grade 3/4 treatment-related adverse event occurred, immunotherapy was suspended and then restarted at the discretion of two or more attending physicians.

NLR was considered the ratio of neutrophil count to lymphocyte count, LMR as lymphocyte count/monocyte count, and CAR as CRP/albumin (mg/g). Overall survival (OS) was defined as the duration from the initiation of treatment to the death of an individual due to any cause or the most recent follow-up date. PFS was defined as the duration from treatment to recurrence or metastasis of the disease. The objective response rate (ORR), encompassing CR and PR, was established as the percentage of individuals who achieved either CR or PR. Meanwhile, the disease control rate (DCR), comprising CR, PR, and SD, was established as the proportion of individuals who either achieved remission or maintained stability during treatment.

### 2.3 Statistical analysis

Statistical analysis was conducted utilizing SPSS v22.0, (IBM Corp., Armonk, NY, USA). Quantitative data with non-normal distribution were expressed by the median and quad spacing [M (P25~P75)]. Mann–Whitney U test was used to determine intergroup differences. Qualitative data were represented by the number of cases (%). Receiver operator characteristic (ROC) curves were generated to compute the area under the curve (AUC) and identify statistically significant variables. The ROC curve analysis was performed to ascertain the maximum Youden indexes for NLR, LMR, and CAR. The associated expression levels of these maximum values served as the cutoff values for NLR, LMR, and CAR. The Kaplan–Meier method was utilized for univariate analysis of survival data, and the Cox proportional risk regression model was employed for multivariate analysis. The nomograms were constructed according to the multivariate analysis index to further predict the survival. Furthermore, the Pearson chi-squared test was used to analyze the association between the inflammatory biomarkers (NLR, LMR, and CAR) and ORR and DCR. Logistic regression was employed to develop a clinical prediction model. A p-value of <0.05 indicated the statistical significance level.

## 3 Results

### 3.1 Patient and disease characteristics

In total, 199 individuals were selected for the final analysis. The median age at baseline was 51 years (44.00–56.00), and the majority of individuals were male (83.42%). Of the 199 individuals, 159 (79.9%) had an ECOG performance score of 0, and 40 (20.1%) had a score of 1. The majority of individuals had cirrhosis (51.26%), mainly secondary to hepatitis B (91.46%). Most of the individuals had a compensatory liver function, with 140 (70.35%) meeting Child–Pugh A, and 59 (29.65%) meeting Child–Pugh B. According to the ALBI grade, 29 cases (14.57%) were ALBI 1, 154 (77.39%) ALBI 2, and 16 (8.04%) ALBI 3. Furthermore, 118 individuals (59.30%) had AFP ≥400 ng/ml. According to the BCLC stage, 132 individuals (66.33%) were BCLC C, and 67 (33.67%) were BCLC B. Twenty-one individuals (10.55%) had lymph node metastasis, and 99 (49.75%) had portal vein tumor thrombus (PVTT). In addition, 35.18% of individuals had changed ICI drugs, and some individuals (74.37%) also used immunoboosters. Most individuals received TACE (79.90%) and HAIC (15.58%) (**Table 1**).

**Table 1:** Patient characteristics.

Variables	All	R	NR	*Z*/*χ*^2^	*p*
Patient number (%)	199 (100)	91 (45.7)	108 (54.3)		
Median age (y, range)	51.00 (44.00–56.00)	52.00 (44.00–57.00)	51.00 (44.00–54.00)	-1.364	0.173
Sex				1.398	0.237
Male	166 (83.42)	79 (86.81)	87 (80.56)		
Female	33 (16.58)	12 (13.19)	21 (19.44)		
ECOG performance score				2.795	0.095
0	159 (79.9)	68 (74.73)	91 (84.26)		
1	40 (20.1)	23 (25.27)	17 (15.74)		
HBV				4.629	0.031
Present	182 (91.46)	79 (86.81)	103 (95.37)		
Absent	17 (8.54)	12 (13.19)	5 (4.63)		
Cirrhosis				3.275	0.070
Present	102 (51.26)	53 (58.24)	49 (45.37)		
Absent	97 (48.74)	38 (41.76)	59 (54.63)		
Child–Pugh class				2.814	0.245
5	67 (33.67)	35 (38.46)	32 (29.63)		
6	73 (36.68)	28 (30.77)	45 (41.67)		
7	59 (29.65)	28 (30.77)	31 (28.7)		
ALBI grade				1.914	0.384
1	29 (14.57)	14 (15.38)	25 (23.15)		
2	154 (77.39)	69 (75.82)	75 (69.44)		
3	16 (8.04)	8 (8.79)	8 (7.41)		
AFP level				5.315	0.024
<400 ng/ml	81 (40.70)	45 (49.45)	36 (33.33)		
≥400 ng/ml	118 (59.30)	46 (50.55)	72 (66.67)		
BCLC stage				1.025	0.311
B	67 (33.67)	34 (37.36)	33 (30.56)		
C	132 (66.33)	57 (62.64)	75 (69.44)		
Lymph node metastasis				6.734	0.009
Present	21 (10.55)	4 (4.4)	17 (15.74)		
Absent	178 (89.45)	87 (95.6)	91 (84.26)		
PVTT				1.126	0.289
Present	99 (49.75)	49 (53.85)	50 (46.3)		
Absent	100 (50.25)	42 (46.15)	58 (53.7)		
ICIs alternation				69.668	<0.001
Present	70 (35.18)	4 (4.4)	66 (61.11)		
Absent	129 (64.82)	87 (95.6)	42 (38.89)		
Combined local treatment				0.004	0.952
TACE	159 (79.9)	76 (83.52)	83 (76.85)		
HAIC	31 (15.58)	15 (16.48)	16 (14.81)		
None	9 (4.52)	0 (0)	9 (8.33)		

R, response group; NR, non-response group; ECOG, Eastern Cooperative Oncology Group; HBV, hepatitis B virus; ALBI, albumin-bilirubin; AFP, alpha fetoprotein; BCLC, Barcelona Clinic Liver Cancer; PVTT, portal vein tumor thrombus; ICIs, immune checkpoint inhibitors; TACE, transcatheter arterial chemoembolization; HAIC, hepatic arterial infusion chemotherapy.

As per the criteria of mRECIST and RECIST1.1, 91 individuals were included in the R group and 108 individuals in the NR group. No remarkable correlations were noted between patient response and baseline characteristics, which encompassed age, gender, ECOG performance score, cirrhosis, Child–Pugh class, ALBI grade, BCLC stage, and PVTT. It is worth noting that the R group had a remarkably reduced number of HBV individuals (p = 0.031), reduced AFP levels (p = 0.014), fewer instances of lymph node metastases (p < 0.01), and higher utilization of immunoboosters (p = 0.018) compared to the NR group (**Table 1**).

### 3.2 Inflammatory biomarkers

**Figure 2:**
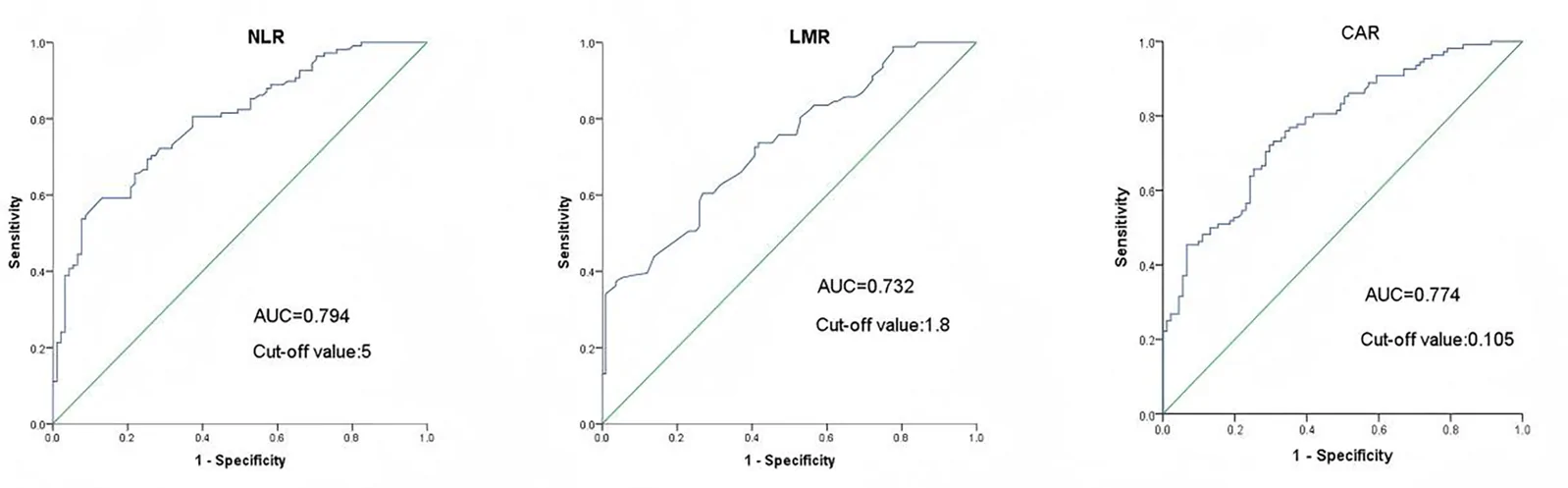
ROC curves for the discriminatory ability of NLR, LMR, and CAR in patients with advanced HCC in the training cohort. ROC, receiver operating characteristic; NLR, neutrophil-lymphocyte ratio; LMR, lymphocyte-monocyte ratio; CAR, C-reactive protein/albumin ratio; HCC, hepatocellular carcinoma.

Pre-treatment blood samples were examined to analyze the relationship between the inflammatory markers (NLR, LMR, and CAR) and survival outcomes in HCC individuals receiving targeted therapy and immunotherapy. In the training cohort, according to the ROC curve analysis, the AUCs of NLR, LMR, and CAR were 0.794 (95% CI 0.733–0.855, p < 0.001), 0.732 (95% CI 0.663–0.801, p < 0.001), and 0.774 (95% CI 0.640–0.846, p = 0.786, p < 0.001), respectively. The cut-off values were 5, 1.8, and 0.105, respectively. Therefore, NLR ≥5, LMR <1.8, and CAR ≥0.105 were used as biomarkers to distinguish the R from the NR groups (**Figure 2**). The NLR of the R group was remarkably reduced in comparison with the NR group (p < 0.001). Additionally, 123 individuals (61.81%) showed an NLR value of <5, of which 64.23% were in the R group. The LMR of the R group was remarkably higher than that of the NR group (p < 0.001), and 38 individuals (19.1%) exhibited LMR ≥1.8, of which 89.47% were in the R group. The CAR in the R group was remarkably decreased than that in the NR group (p < 0.001), and 94 individuals (47.24%) showed CAR <0.105, of which 68.09% were in the R group (**Table 2**).

**Table 2:** Inflammatory biomarkers characteristics.

Variables	All	R	NR	*Z*/*χ*^2^	*p*
Median neutrophil count × 10^9/L (range)	5.52 (4.31–6.59)	5.00 (4.21–5.90)	6.25 (4.50–7.46)	-4.187	<0.001
Median lymphocyte count × 10^9/L (range)	1.16 (1.00–1.30)	1.19 (1.05–1.40)	1.13 (0.98–1.26)	-2.873	0.004
Median monocyte count × 10^9/L (range)	0.76 (0.62–0.93)	0.78 (0.58–0.92)	0.74 (0.65–0.96)	-1.100	0.271
Median albumin value g/L (range)	34.80 (32.60–37.20)	35.40 (32.60–37.40)	34.45 (32.63–36.98)	-0.714	0.475
Median CRP value mg/L (range)	3.70 (2.80–5.60)	3.00 (1.90–4.00)	4.40 (3.40–6.40)	-6.665	<0.001
NLR				44.410	<0.001
<5	123.00 (61.81)	79 (86.81)	44 (40.74)		
≥5	76 (38.19)	12 (13.19)	64 (59.26)		
LMR				36.217	<0.001
<1.8	161 (80.9)	57 (62.64)	104 (96.3)		
≥1.8	38 (19.1)	34 (37.36)	4 (3.70)		
CAR				35.879	<0.001
<0.105	94 (47.24)	64 (70.33)	30 (27.78)		
≥0.105	105 (52.76)	27 (29.67)	78 (72.22)		

R, response group; NR, non-response group; CRP, C-reactive protein; NLR, neutrophil-lymphocyte ratio; LMR, lymphocyte-monocyte ratio; CAR, CRP-albumin ratio.

The baseline clinicopathologic features depicted remarkable variation between the NLR stratified groups, with individuals with NLR ≥5 having a higher risk of HBV infection (p = 0.039), higher AFP value (p < 0.001), more portal lymph node metastases (p < 0.001), and more PVTT (p = 0.017). LMR-stratified individuals showed significant differences in AFP values (p = 0.042). Furthermore, there were significant differences in liver cirrhosis (p = 0.001), ALBI grade (p < 0.01), and AFP value (p = 0.012) among CAR-stratified individuals (**Table 3**).

**Table 3:** Relationship between inflammatory status and baseline clinicopathological characteristics.

Variables	NLR			LMR			CAR		
	<5	≥5	*p*	<1.8	≥1.8	*p*	<0.105	≥0.105	*p*
ECOG performance score (0/1)	94/29 (76.42) (23.58)	65/11 (85.53) (14.47)	0.119	130/31 (80.75) (19.25)	29/9 (76.32) (23.68)	0.540	73/21 (77.66) (22.3)	86/19 (81.9) (18.1)	0.456
HBV (absent/present)	13/110 (10.57) (89.43)	2/74 (2.63) (97.37)	0.039	10/151 (6.21) (93.79)	5/33 (13.16) (86.84)	0.145	10/84 (10.64) (89.36)	5/100 (4.76) (95.24)	0.117
Cirrhosis (absent/present)	61/62 (49.59) (50.41)	39/37 (51.32) (48.68)	0.813	85/76 (52.8) (47.2)	15/23 (39.47) (60.53)	0.140	59/35 (62.77) (37.23)	41/64 (39.05) (60.95)	0.001
Child–Pugh class (5/6/7)	44/38/41 (35.77) (30.89) (33.33)	23/35/18 (30.26) (46.05) (23.68)	0.089	53/59/49 (32.92) (36.65) (30.43)	14/14/10 (36.84) (36.84) (26.32)	0.854	32/35/27 (34.04) (37.23) (28.72)	35/38/32 (33.33) (36.19) (30.48)	0.964
ALBI grade (1/2/3)	19/95/9 (15.45) (77.24) (7.32)	10/59/7 (13.16) (77.63) (9.21)	0.827	26/121/14 (16.15) (75.16) (8.7)	3/33/2 (7.89) (86.84) (5.26)	0.297	12/80/2 (12.77) (85.11) (2.13)	17/74/14 (16.19) (70.48) (13.33)	0.009
AFP (<400/≥400)	65/58 (52.85) (47.15)	16/60 (21.05) (78.95)	<0.001	60/101 (37.27) (62.73)	21/17 (55.26) (44.74)	0.042	47/47 (50) (50)	34/71 (32.38) (67.62)	0.012
BCLC stage (B/C)	45/78 (36.59) (63.41)	22/54 (28.95) (71.05)	0.268	51/110 (31.68) (68.32)	16/22 (42.11) (57.89)	0.221	34/60 (36.17) (63.83)	33/72 (31.43) (68.57)	0.480
Lymph node metastasis (absent/present)	119/4 (96.75) (3.25)	59/17 (77.63) (22.37)	<0.001	141/20 (87.58) (12.42)	37/1 (97.37) (2.63)	0.077	86/8 (91.49) (8.51)	92/13 (87.62) (12.38)	0.375
PVTT (absent/present)	70/53 (56.91) (43.09)	30/46 (39.47) (60.53)	0.017	79/82 (49.07) (50.93)	21/17 (55.26) (44.74)	0.492	44/50 (46.81) (53.19)	56/49 (53.33) (46.67)	0.358

NLR, neutrophil-lymphocyte ratio; LMR, lymphocyte-monocyte ratio; CAR, C-reactive protein/albumin ratio; ECOG, Eastern Cooperative Oncology Group; HBV, hepatitis B virus; ALBI, albumin-bilirubin; BCLC, Barcelona Clinic Liver Cancer; PVTT, portal vein tumor thrombus.

### 3.3 Survival analysis

**Figure 3:**
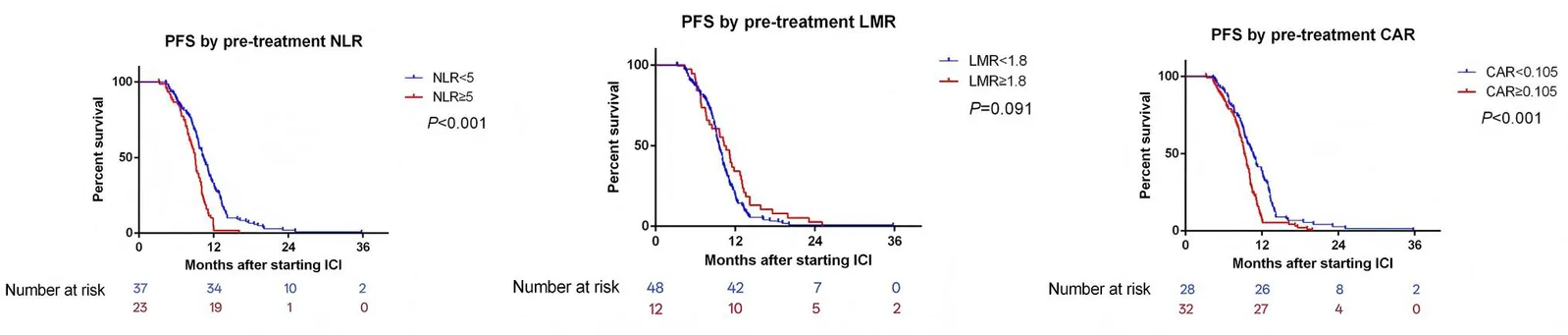
KM curves of PFS for NLR, LMR, and CAR in the verification cohort. KM, Kaplan–Meier; NLR, neutrophil-lymphocyte ratio; LMR, lymphocyte-monocyte ratio; CAR, C-reactive protein/albumin ratio; PFS, progression-free survival.

In the verification cohort, according to the Kaplan–Meier survival curve, NLR ≥5 (10.4 vs. 9 mo, HR 2.146, 95% CI 1.564–2.946, p < 0.001) and CAR ≥0.105 (10.6 vs. 9.2 mo, HR 1.791, 95% CI 1.329–2.413, p < 0.001)remarkably shortened the mPFS in the enrolled individuals (**Figure 3**).

The univariate analysis showed that Child–Pugh class (HR 1.416, 95% CI 1.032–1.943, p = 0.031), ALBI grade (HR 1.543, 95% CI 1.027–2.319, p = 0.037), and AFP (HR 1.554, 95% CI 1.152–2.098, p < 0.01) were linked to poor PFS (**Table 4**). These related single factors were selected for multivariate analysis. The acquired data implied that NLR ≥5 (HR 1.819, 95% CI 1.291–2.562, p = 0.001) and CAR ≥0.105 (HR 1.609, 95% CI 1.164–2.223, p = 0.004) remained independent prognostic factors for PFS, including ALBI grade (HR 1.619, 95% CI 1.057–2.480, p = 0.027) and Child–Pugh (HR 1.420, 95% CI 1.030–1.958, p = 0.032) (**Table 4**).

**Table 4:** Univariate and multivariate Cox regression model assessing for PFS in the verification cohort.

Variables	(*n* = 60)	Univariate analysis		Multivariate analysis	
		HR (95% CI)	*p*	HR (95% CI)	*p*
ECOG performance score (0/1)	48/12	0.974 (0.683–1.387)	0.882		
HBV (absent/present)	5/55	1.729 (0.999–2.99)	0.050		
Cirrhosis (absent/present)	30/30	1.148 (0.861–1.529)	0.348		
Child-Pugh class (5-6/7)	42/18	1.416 (1.032–1.943)	0.031	1.420 (1.030–1.958)	0.032
ALBI grade (1/2-3)	9/51	1.543 (1.027–2.319)	0.037	1.619 (1.057–2.480)	0.027
AFP (<400/≥400)	24/36	1.554 (1.152–2.098)	0.004	1.286 (0.932–1.773)	0.126
BCLC stage (B/C)	20/40	0.973 (0.719–1.317)	0.857		
PVTT (absent/present)	30/30	1.241 (0.933–1.649)	0.138		
NLR (<5/≥5)	37/23	2.146 (1.564–2.946)	<0.001	1.819 (1.291–2.562)	0.001
LMR (<1.8/≥1.8)	48/12	0.732 (0.51–1.051)	0.091		
CAR (<0.105/≥0.105)	28/32	1.791 (1.329–2.413)	<0.001	1.609 (1.164–2.223)	0.004

PFS, progression-free survival; HR, hazard ratio; CI, confidence interval; ECOG, Eastern Cooperative Oncology Group; HBV, hepatitis B virus; ALBI, albumin-bilirubin; AFP, alpha fetoprotein; BCLC, Barcelona Clinic Liver Cancer; PVTT, portal vein tumor thrombus; NLR, neutrophil-lymphocyte ratio; LMR, lymphocyte-monocyte ratio; CAR, C-reactive protein/albumin ratio.

**Figure 4:**
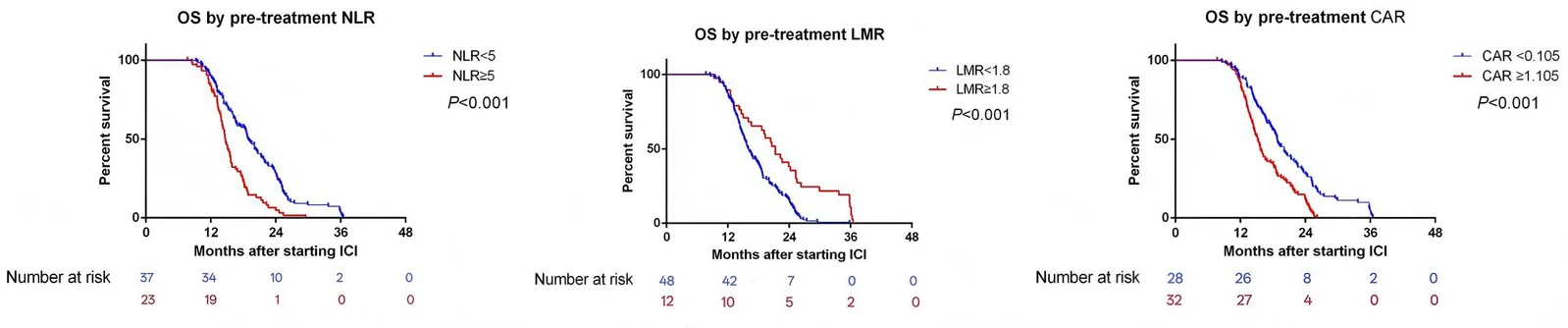
KM curves of OS for NLR, LMR, and CAR in the verification cohort. KM, Kaplan–Meier; NLR, neutrophil-lymphocyte ratio; LMR, lymphocyte-monocyte ratio; CAR, C-reactive protein/albumin ratio; OS, overall survival.

In the verification cohort, the Kaplan–Meier survival curve indicated that individuals with NLR ≥5 had remarkably reduced OS than those with NLR <5 (18.9 vs. 14.7 mo, HR 2.268, 95% CI 1.654–3.112 p < 0.001). Similarly, LMR <1.8 (21.3 vs. 16 mo, HR 0.430, 95% CI 0.286–0.648, p < 0.001) and CAR ≥0.105 (18.8 vs. 15.2 mo, HR 2.021, 95% CI 1.481–2.757, p < 0.001) reported shorter OS (**Figure 4**).

In the univariate analysis, other parameters linked to poor OS were HBV infection (HR 1.849, 95% CI 1.046–3.269, p = 0.034), cirrhosis (HR 1.354, 95% CI 1.009–1.817, p = 0.043), and high AFP value (HR 1.603, 95% CI 1.182–2.174, p < 0.01). These correlated single factors were selected for multivariate analysis. NLR ≥5 (HR 1.718, 95% CI 1.215–2.428, p = 0.002), LMR <1.8 (HR 0.547, 95% CI 0.355–0.843, p = 0.006), and CAR ≥0.105 (HR 1.474, 95% CI 1.043–2.081, p = 0.028) remained poor predictors of OS (**Table 5**).

**Table 5:** Univariate and multivariate Cox regression model assessing for OS in the verification cohort.

Variable	(*n* = 60)	Univariate analysis		Multivariate analysis	
		HR (95% CI)	*P*	HR (95% CI)	*P*
ECOG performance score (0/1)	48/12	1.248 (0.872–1.787)	0.225		
HBV (absent/present)	5/55	1.849 (1.046–3.269)	0.034	1.546 (0.865–2.762)	0.142
Cirrhosis (absent/present)	30/30	1.354 (1.009–1.817)	0.043	1.260 (0.911–1.745)	0.163
Child–Pugh class (5-6/7)	42/18	1.159 (0.84–1.599)	0.368		
ALBI grade (1/2-3)	9/51	0.892 (0.594–1.34)	0.582		
AFP (<400/≥400)	24/36	1.603 (1.182–2.174)	0.002	1.128 (0.814–1.563)	0.470
BCLC stage (B/C)	20/40	1.163 (0.850–1.593)	0.345		
PVTT (absent/present)	30/30	1.252 (0.936–1.676)	0.130		
NLR (<5/≥5)	37/23	2.268 (1.654–3.112)	<0.001	1.718 (1.215–2.428)	0.002
LMR (<1.8/≥1.8)	48/12	0.430 (0.286–0.648)	<0.001	0.547 (0.355–0.843)	0.006
CAR (<0.105/≥0.105)	28/32	2.021 (1.481–2.757)	<0.001	1.474 (1.043–2.081)	0.028

OS, overall survival; HZ, hazard ratio; CI, confidence interval; ECOG, Eastern Cooperative Oncology Group; HBV, hepatitis B virus; ALBI, albumin-bilirubin; AFP, alpha fetoprotein; BCLC, Barcelona Clinic Liver Cancer; PVTT, portal vein tumor thrombus; NLR, neutrophil-lymphocyte ratio; LMR, lymphocyte-monocyte ratio; CAR, C-reactive protein/albumin ratio.

### 3.4 Creation and validation of nomograms

**Figure 5:**
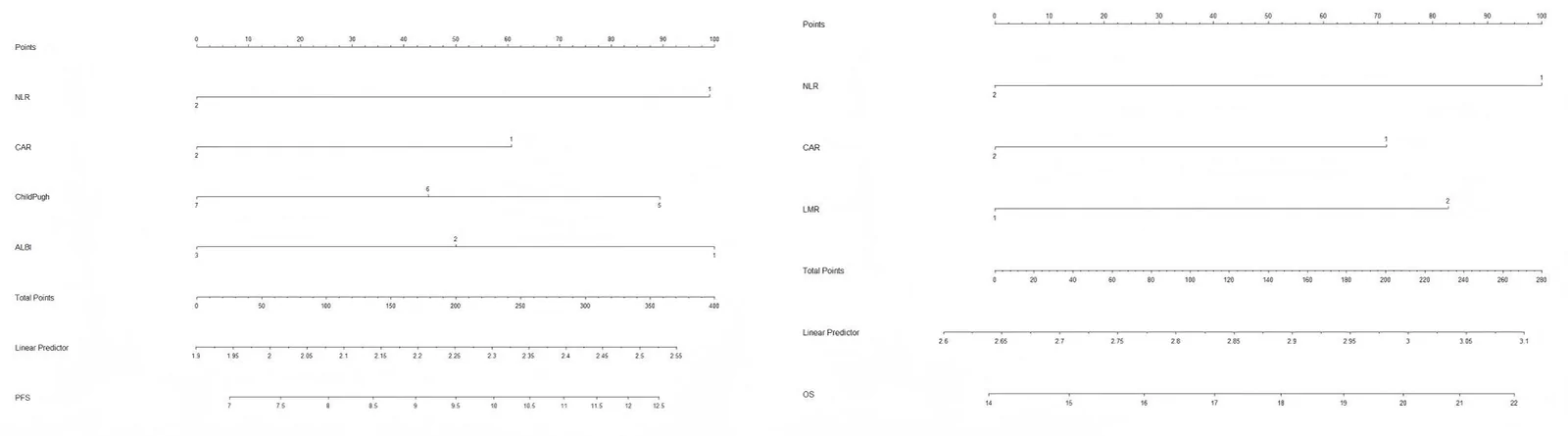
Establishment of PFS and OS nomograms in the verification cohort.

Hence, we utilized relevant factors from multivariate analysis to construct nomograms to predict patient survival in the verification cohort (**Figure 5**).

**Figure 6:**
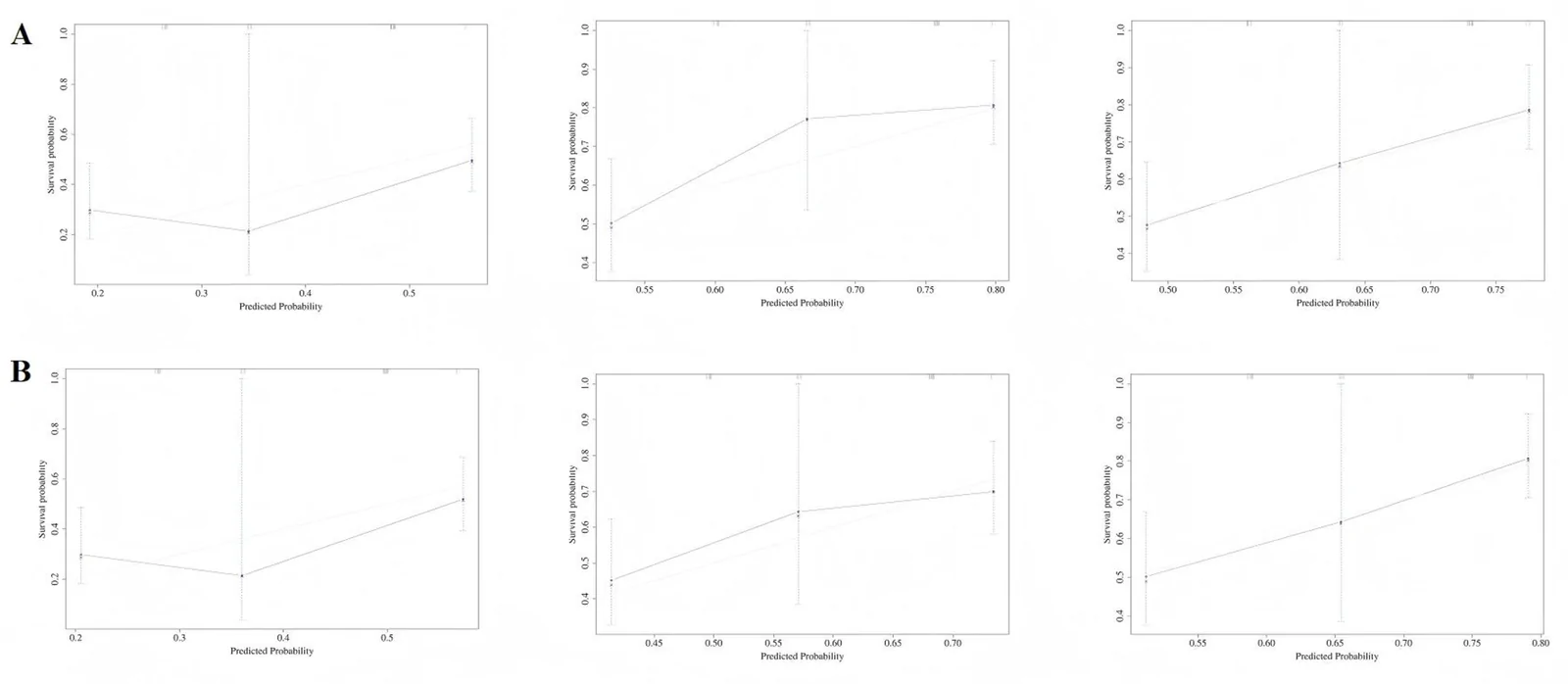
The calibration curves for predicting OS at 1-year, 2-year and 3-year **(A)**. The calibration curves for predicting PFS at 1-year, 2-year and 3-year **(B)**.

**Figure 7:**
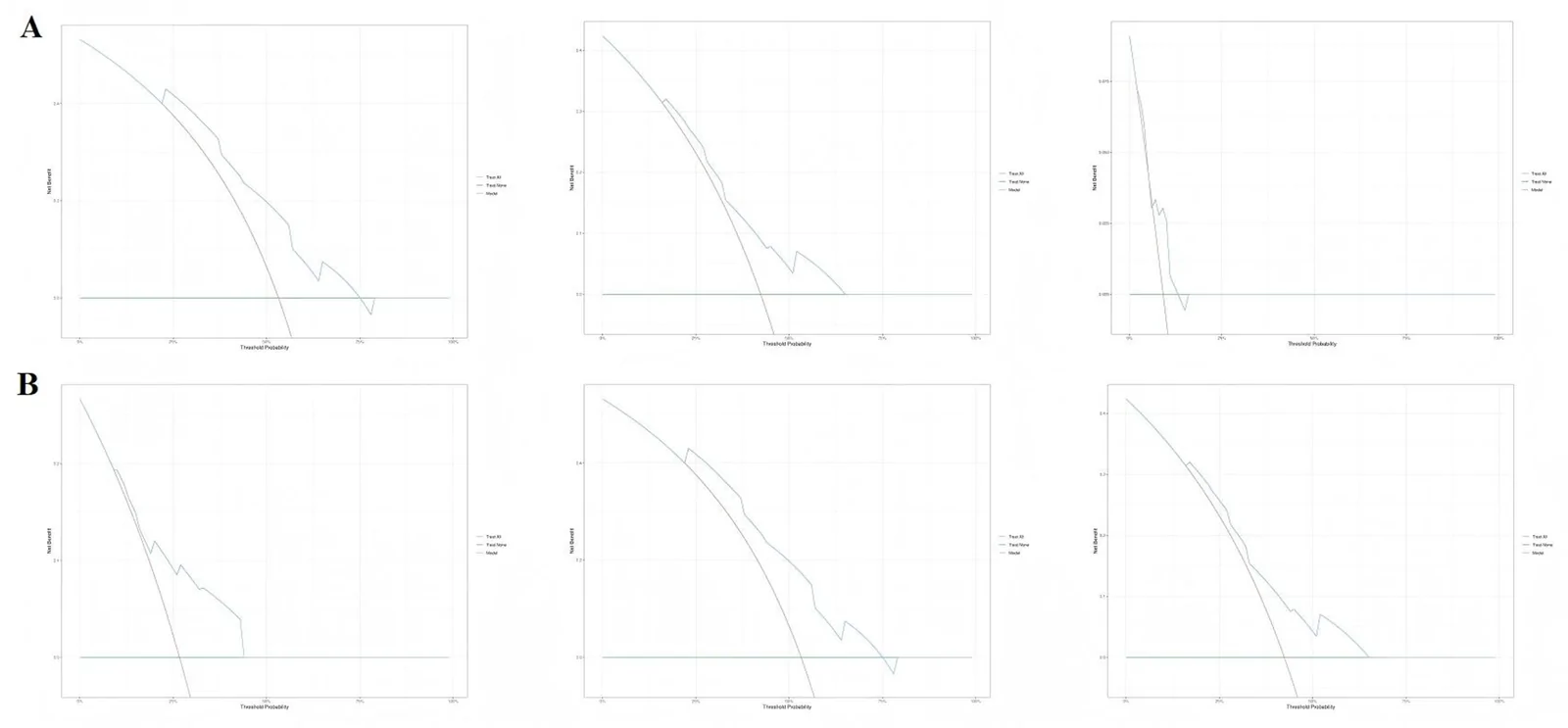
Decision curves of the nomogram predicting OS at 1-year, 2-year and 3-year **(A)**. Decision curves of the nomogram predicting PFS at 1-year, 2-year and 3-year **(B)**.

The internal validation of the nomogram model was conducted using the Bootstrap repeated sampling method. After 100 repetitions of Bootstrap self-sampling, the calibration curves were obtained, and the trends of the two curves were basically consistent, showing strong consistency (**Figure 6**). The decision curve of the nomogram can predict the 1-year, 2-year, and 3-year OS rates and PFS rates of the validation group (**Figure 7**). This result highlights the robust clinical predictive ability of the nomogram.

### 3.5 Response analysis

**Figure 8:**
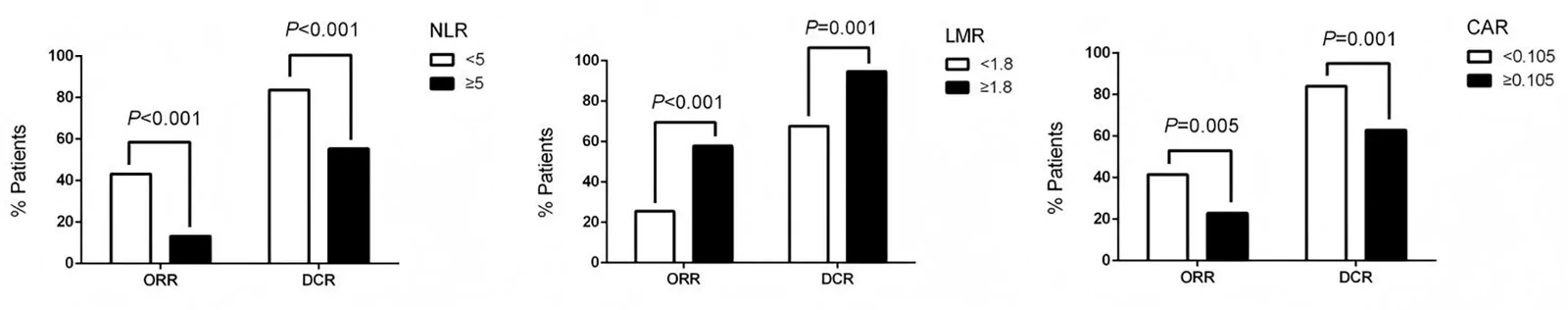
Histograms demonstrating the differences in inflammatory biomarkers according to response in the verification cohort.

In the verification cohort, the ORR was recorded to be 31.66% in 63 individuals, and the DCR was 72.86% in 145 individuals. Individuals with NLR <5 were significantly more likely to achieve ORR than those with NLR ≥5 (43.09% vs. 13.16%, p < 0.001). Similarly, LMR ≥1.8 (57.89% vs. 25.47%, p < 0.001) and CAR <0.105 (41.49% vs. 22.86%, p < 0.01) were linked to a higher proportion of ORR (**Figure 8**).

In addition, in logistic univariate regression analysis, cirrhosis (OR 0.441, 95% CI 0.238–0.815, p = 0.009) and low ALBI grade (OR 0.062, 95% CI 0.008–0.468, p = 0.007) were linked to worse ORR. These related single factors were selected for multivariate analysis. NLR ≥5 (OR 4.729, 95% CI 2.036–10.98, p < 0.001), LMR <1.8 (OR 0.487, 95% CI 0.212–1.119, p = 0.090), CAR ≥0.105 (OR 2.661, 95% CI 1.193–5.934, p = 0.017) cirrhosis (OR 0.259, 95% CI 0.115–0.581, p = 0.001) and low ALBI grade (OR 0.048, 95% CI 0.006–0.38, p = 0.004) remained independent prognostic factors for ORR (**Table 6**).

**Table 6:** Univariate and multivariate logistic regression model assessing for ORR in the verification cohort.

Variables	(*n* = 60)	Univariate analysis		Multivariate analysis	
		*OR* (95% CI)	*P*	*OR* (95% CI)	*P*
ECOG performance score (0/1)	48/12	0.721 (0.349~1.487)	0.375		
HBV (absent/present)	5/55	2.681 (0.927~7.755)	0.069		
Cirrhosis (absent/present)	30/30	0.441 (0.238~0.815)	0.009	0.259 (0.115~0.581)	0.001
Child-Pugh class (5-6/7)	42/18	0.965 (0.503~1.853)	0.915		
ALBI grade (1/2-3)	9/51	0.062 (0.008~0.468)	0.007	0.048 (0.006~0.38)	0.004
AFP (<400/≥400)	24/36	1.139 (0.621~2.087)	0.674		
BCLC stage (B/C)	20/40	1.331 (0.713~2.484)	0.369		
Lymph node metastasis	30/30	3.051 (0.864~10.768)	0.083		
PVTT (absent/present)	37/23	0.781 (0.429~1.421)	0.418		
NLR (<5/≥5)	48/12	4.997 (2.349~10.63)	<0.001	4.729 (2.036~10.98)	<0.001
LMR (<1.8/≥1.8)	28/32	0.248 (0.119~0.518)	<0.001	0.487 (0.212~1.119)	0.090
CAR (<0.105/≥0.105)	48/12	2.393 (1.296~4.418)	0.005	2.661 (1.193~5.934)	0.017

ORR, objective response rate; OR, odds ratio; CI, confidence interval; ECOG, Eastern Cooperative Oncology Group; HBV, hepatitis B virus; ALBI, albumin-bilirubin; AFP, alpha fetoprotein; BCLC, Barcelona Clinic Liver Cancer; PVTT, portal vein tumor thrombus; NLR, neutrophil-lymphocyte ratio; LMR, lymphocyte-monocyte ratio; CAR, C-reactive protein/albumin ratio.

**Figure 9:**
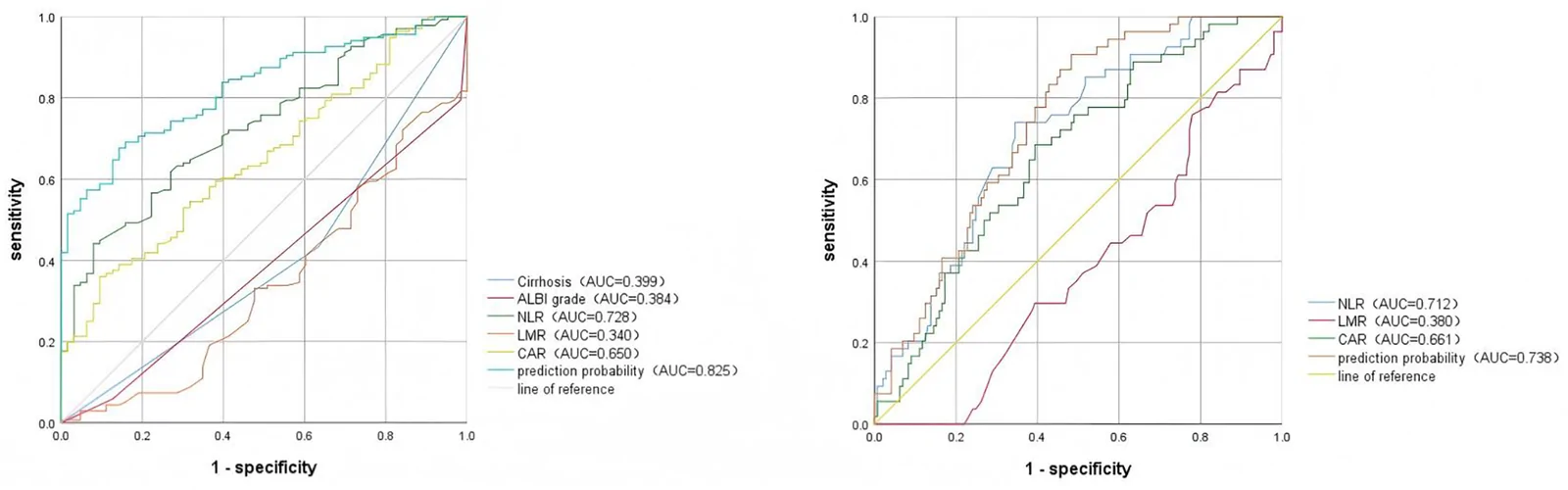
ROC curves for the discriminatory ability of prediction probability in ORR and DCR. ROC, receiver operating characteristic; ORR, objective response rate; DCR, disease control rate.

The regression equation is as follows: logit(p) = 0.801 + 0.568 × NLR - 0.145 × LMR + 11.352 × CAR -1.025 × cirrhosis -1.479 × ALBI. The AUC value of prediction probability was 0.825, and the prediction performance of the model was superior to that of NLR (AUC = 0.794), LMR (AUC = 0.732), and CAR (AUC = 0.774) (**Figure 9**).

The proportion of individuals with NLR <5 who achieved DCR was remarkably elevated than those with NLR ≥5 (83.74% vs. 55.26%, p < 0.001). Similarly, LMR ≥1.8 (94.74% vs. 67.7%, p = 0.001) and CAR <0.105 (84.04% vs. 62.86%, p = 0.001) were linked to a higher proportion of DCR (**Figure 8**).

In addition, in logistic univariate regression analysis, high AFP (OR 2.454, 95% CI 1.23–4.896, p = 0.011) was linked to poor ORR. These related single factors were selected for multivariate analysis. NLR ≥5 (OR 2.607, 95% CI 1.274–5.334, p = 0.009), LMR <1.8 (OR 0.191, 95% CI 1.274–5.334, p = 0.009), 95% CI 0.043–0.858, p = 0.031), and CAR ≥0.105 (OR 2.134, 95% CI 1.032–4.411, p = 0.041) remained independent prognostic factors for DCR (**Table 7**).

**Table 7:** Univariate and multivariate logistic regression model assessing for DCR in the verification cohort.

Variables	(*n* = 60)	Univariate analysis		Multivariate analysis	
		*OR* (95% CI)	*p*	*OR* (95% CI)	*p*
ECOG performance score (0/1)	48/12	0.408 (0.161~1.036)	0.059		
HBV (absent/present)	5/55	5.664 (0.726~44.161)	0.098		
Cirrhosis (absent/present)	30/30	0.827 (0.442~1.547)	0.552		
Child-Pugh class (5-6/7)	42/18	0.999 (0.504~1.980)	0.997		
ALBI grade (1/2-3)	9/51	0.663 (0.287~1.536)	0.338		
AFP (<400/≥400)	24/36	2.454 (1.23~4.896)	0.011	1.613 (0.755~3.447)	0.217
BCLC stage (B/C)	20/40	1.646 (0.82~3.302)	0.161		
Lymph node metastasis	30/30	1.766 (0.688~4.532)	0.237		
PVTT (absent/present)	37/23	0.747 (0.399~1.399)	0.362		
NLR (<5/≥5)	48/12	4.169 (2.158~8.055)	<0.001	2.607 (1.274~5.334)	0.009
LMR (<1.8/≥1.8)	28/32	0.116 (0.027~0.502)	0.004	0.191 (0.043~0.858)	0.031
CAR (<0.105/≥0.105)	48/12	3.112 (1.578~6.139)	0.001	2.134 (1.032~4.411)	0.041

DCR, disease control rate; OR, odds ratio; CI, confidence interval; ECOG, Eastern Cooperative Oncology Group; HBV, hepatitis B virus; ALBI, albumin-bilirubin; AFP, alpha fetoprotein; BCLC, Barcelona Clinic Liver Cancer; PVTT, portal vein tumor thrombus; NLR, neutrophil-lymphocyte ratio; LMR, lymphocyte-monocyte ratio; CAR, C-reactive protein/albumin ratio.

The regression equation is as follows: logit(p) = -1.908 + 0.3 × NLR -0.725 × LMR + 4.121 × CAR. The AUC value of prediction probability was 0.738, and the prediction performance of the model was superior to that of LMR alone (AUC = 0.732) (**Figure 9**).

## 4 Discussion

Combined targeted therapy and immunotherapy greatly prolonged the survival time of HCC individuals (20). Nevertheless, the ORR of atezolizumab + bevacizumab was merely approximately 30%, with around 5%–30% of individuals developing grade ≥3 immune-related adverse events (21). Therefore, there is an urgent need to determine the clinical or biological indicators that can be used to predict the efficacy of ICI combination therapy. Although there are existing predictive biomarkers, their effects on HCC are relatively poor. The expression level of PD-L1 on tumor-infiltrating cells and/or tumor cells has a prognostic effect on HCC (22). Nonetheless, there is a dearth of supporting evidence regarding its predictive utility in individuals undergoing combination therapy with ICIs. This can primarily be attributed to the challenges posed by the spatial and temporal heterogeneity of its expression and the absence of established protocols for its detection and interpretation (9). Furthermore, it is rare to detect high TMB and microsatellite instability in cases of HCC (23). Genomic studies have revealed genetic signatures that may be linked to ICIs (24) and proposed methods for immunoclassification based on molecular signatures (25). However, prospective clinical studies are lacking. With the ongoing phase III clinical trials of ICI combination therapy, combined targeted therapy and immunotherapy have become the first-line treatment for unresectable HCC.

Several studies have shown the importance of inflammation in cancer progression (26). HCC represents a typical inflammatory ailment triggered by underlying conditions like cirrhosis, fibrosis, and chronic liver inflammation. This inflammation triggers a reduction in the generation of both immune and inflammatory cells by attracting pro-inflammatory cytokines and regulatory T lymphocytes. This, in turn, activates downstream signaling pathways involving STAT3 and NF-κB, resulting in an imbalanced host immune response and consequently causing a loss of response to chemotherapy (27). Over the past few years, numerous investigations have delved into the effect of inflammatory markers on individuals undergoing various treatment approaches for HCC. For instance, a study suggested that preoperative NLR and platelet-to-lymphocyte ratio (PLR) are linked to poor prognosis of resectable HCC. NLR and PLR are predictors of efficacy response after HAIC treatment of HCC, and SII (Systemic Inflammation Index) can predict the prognosis of individuals after radical resection of HCC (13).

Serum inflammatory biomarkers are low-cost and easy to obtain. In the present study, these biomarkers helped to identify HCC individuals with good prognosis after ICI treatment. We found the following clinical associations: 1) NLR <5, LMR ≥1.8, and CAR <0.105 were associated with better outcomes and could serve as useful predictors in HCC patients receiving ICI therapy; 2) higher NLR, lower LMR, and higher CAR were associated with poorer OS in HCC individuals treated with ICIs; 3) higher NLR, lower LMR, and higher CAR were associated with a lower ORR in HCC individuals receiving ICIs.

It is important to note that our study did not include immune-cell subset analyses or cytokine profiling. Therefore, the biological mechanisms underlying these clinical associations remain speculative and are discussed below based on evidence from the existing literature, rather than direct experimental validation in our cohort. Previous studies have suggested that the dysfunction of CD8+ T cells and the overexpression of inhibitory T cells may lead to the escape of tumors from the immune system (28). Neutrophils and platelets are thought to be involved in the immune escape of malignant cells by producing cytokines, such as IL-18, VEGF, and platelet-derived growth factor (26). In addition, circulating neutrophils may contribute to cancer growth and progression through neutrophil extracellular trap (NET) formation (29). Furthermore, M2 macrophages have pro-tumor activities that promote the proliferation, migration, angiogenesis, and immunosuppression of liver cancer cells; accordingly, M2 macrophages are linked to the poor prognosis of HCC (30). Among individuals with HCC, thrombocytopenia is often an indicator of hypersplenism secondary to portal hypertension, potentially affecting the prognostic value of an elevated PLR for poor prognosis. The levels of albumin and CRP depend on the synthesis capacity of the liver; therefore, CAR is closely related to liver function. The prognostic value of CAR should be carefully evaluated in the context of liver dysfunction. High CAR levels are thought to reflect the upregulation of proinflammatory cytokines (IL-6 and IL-1), which are linked to neoplastic inflammation and anti-cancer drug resistance (31). These mechanistic hypotheses, while plausible, require dedicated translational studies to be confirmed.

Furthermore, the study noted that not all individuals who achieved ORR had a good survival prognosis. Lencioni et al. analyzed the data from the BRISK-PS phase III randomized controlled clinical trial and found that ORR was closely related to OS (32). On the contrary, another study conducted in 2021 found that although there was no statistical difference in ORR between the treatment and control groups, there was a statistically significant variation in OS (33) between the two groups. Initially, it was believed that there was a direct link between tumor progression and death, especially decades ago when most individuals with HCC were diagnosed at an advanced stage. Today, however, diagnosis is often made at an early asymptomatic stage, with multiple treatment options available, and even if the disease progresses, there is a greater chance of survival. Overall, the association between HCC progression and death is very ambiguous. Most HCC individuals die from liver failure rather than tumor progression.

This study has several strengths, including the first report of the prognostic value of CAR in HCC patients receiving ICI therapy, and the exclusion of patients with acute inflammation or those using confounding medications. High NLR and CAR predicted poor prognosis, while high LMR predicted better outcomes. However, several limitations should be acknowledged. First, the retrospective, single-center design with a modest sample size may introduce selection bias and limit generalizability. Second, treatment regimens were heterogeneous, including varied locoregional therapies and ICI switching in 35.18% of patients, mostly after progression and driven by individual circumstances. Local therapies did not differ significantly between groups (p = 0.952), and baseline biomarkers reflect host status rather than treatment effects, so the impact on our primary findings is likely limited. Third, and importantly, we did not perform immune-cell subset analyses or cytokine measurements; therefore, the biological mechanisms discussed above remain speculative and cannot be directly validated by our data. Additional limitations include the lack of external validation, the absence of longitudinal biomarker measurements, and the unavailability of data on tumor mutational burden or PD-L1 expression. Prospective multicenter studies with translational correlative analyses are warranted to confirm these findings and to elucidate the mechanistic basis of inflammatory biomarkers in HCC patients receiving immunotherapy.

## 5 Conclusions

NLR, LMR, and CAR are potential predictive biomarkers of targeted therapy and immunotherapy efficacy in individuals with advanced HCC. However, with the emergence of new immunotherapy strategies such as CAR-T, further studies investigating the complex interactions between systemic inflammation and the immune system are warranted.

## Data Availability

The original contributions presented in the study are included in the article/supplementary material. Further inquiries can be directed to the corresponding author.
